# Applied evolutionary psychology in moral education: a quasi-experimental study exploring the longitudinal associations of compassion training with emotion regulation and prosocial ethical development

**DOI:** 10.3389/fpsyg.2026.1916084

**Published:** 2026-08-28

**Authors:** Zening Zhang

**Affiliations:** School of Marxism, Beijing Foreign Studies University, Beijing, China

**Keywords:** applied evolutionary psychology, compassion training, emotion regulation, higher education, longitudinal mediation, moral education, moral identity, prosocial ethical development

## Abstract

**Introduction:**

Moral education increasingly requires psychologically realistic approaches that explain how students transform moral knowledge into stable prosocial action. Drawing on applied evolutionary psychology, this longitudinal study examined whether compassion-training engagement in moral education was associated with students’ emotion regulation, moral identity integration, and prosocial ethical development across one academic semester.

**Methods:**

A total of 458 undergraduates enrolled in moral education modules in Sino-foreign cooperative university contexts completed baseline, mid-semester, and endline assessments. The intervention comprised 8 weekly sessions facilitated by trained instructors, focusing primarily on suffering awareness and action planning. A parallel mediation model was used to test whether emotion regulation and moral identity integration transmitted the association between compassion-training engagement and prosocial ethical development.

**Results:**

Compassion-training engagement was positively associated with emotion regulation (β = 0.41, *p* < 0.001), moral identity integration (β = 0.36, *p* < 0.001), and prosocial ethical development (β = 0.19, *p* < 0.001). Both indirect paths were significant: through emotion regulation [β = 0.14, 95% CI (0.09, 0.20)] and moral identity integration [β = 0.10, 95% CI (0.06, 0.16)].

**Discussion:**

The findings suggest that compassion-training engagement may support moral education by facilitating students’ shift from threat-reactive responses toward affiliative care, reflective self-regulation, and ethically consistent action.

## 1 Introduction

Moral education often assumes that students who understand ethical principles will also act on them. Psychological research, however, shows that moral behavior depends not only on reasoning but also on emotion, motivation, identity, and the capacity to regulate responses under social pressure (Haidt, 2001; Decety and Cowell, 2014). Daily moral conduct is shaped by everyday social encounters rather than only by formal ethical reasoning, and prosocial behavior is best understood through multilevel influences that include affect, motivation, norms, relationships, and context (Penner et al., 2005; Hofmann et al., 2014). This distinction is especially important in higher education contexts where students negotiate academic competition, peer comparison, intercultural communication, and collective responsibility. In such settings, moral education must do more than transmit normative content; it must cultivate the psychological capacities that make ethical action possible in everyday life.

Educational psychology, when informed by foundational concepts from compassion theory and affiliative motivation, provides a useful framework for this task. From an evolutionary perspective, compassion is not merely a pleasant emotion or a general attitude of kindness. It is an affiliative motivational system that orients individuals toward the suffering of others, supports approach rather than avoidance, and helps stabilize cooperative social life (Goetz et al., 2010; Gilbert, 2014). Evidence from comparative and developmental work also links empathy-related responding to prosocial behavior and suggests that caregiving and cooperation rely on conserved social-motivational systems (Eisenberg et al., 2010; Decety et al., 2016). The theory of morality-as-cooperation similarly suggests that many moral norms are organized around recurrent problems of social cooperation, including helping, fairness, group commitment, and conflict resolution (Curry et al., 2019). Moral education informed by this perspective therefore asks how educational practice can activate caring motivation, regulate threat-based reactions, and translate moral concern into prosocial ethical behavior.

Compassion training is one promising educational approach. Prior intervention studies indicate that structured compassion cultivation can increase compassion for self and others, reduce emotional suppression and worry, and strengthen altruistic or helping behavior (Jazaieri et al., 2013, 2014; Weng et al., 2013; Kirby et al., 2017). Short-term compassion training has been shown to increase prosocial behavior, and neurocognitive studies indicate that compassion practice can alter positive affect, empathic accuracy, stress responses, and neural responses relevant to suffering and care (Pace et al., 2009; Leiberg et al., 2011; Mascaro et al., 2013; Klimecki et al., 2013, 2014). The distinction between empathic distress and compassion is important because compassion is an approach-oriented care response rather than distress sharing alone (Singer and Klimecki, 2014). Meta-analytic evidence further suggests that kindness-based, loving-kindness, compassion, and mindfulness-related practices can improve wellbeing, positive emotion, compassion, empathy, and prosocial outcomes, although effect sizes vary by method and outcome (Galante et al., 2014; Zeng et al., 2015; Luberto et al., 2018; Donald et al., 2019; Rupprecht et al., 2023; Tickell et al., 2024). Recent systematic reviews underscore that while these interventions consistently foster interpersonal warmth, their longitudinal integration into formal academic curricula remains empirically underexplored (Rupprecht et al., 2023). These effects are theoretically relevant to moral education because compassion training combines attention to suffering, perspective-taking, affect regulation, reflective practice, and action-oriented care. It may therefore provide a bridge between moral cognition and moral conduct.

Two psychological mechanisms are especially important in this bridge. The first is emotion regulation, defined as the processes through which individuals influence the emotions they have, when they have them, and how they experience and express them (Gross, 1998, 2015). In moral contexts, emotion regulation can reduce impulsive anger, defensive avoidance, shame, or punitive responses, allowing students to respond to conflict and suffering with greater perspective and care. The second is moral identity integration, or the degree to which moral traits such as fairness, responsibility, and compassion become central to the self-concept (Aquino and Reed, 2002; Reed and Aquino, 2003; Hardy and Carlo, 2011; Hertz and Krettenauer, 2016). Moral identity and self-regulation are linked to prosocial and antisocial developmental outcomes, suggesting that moral education should connect internalized values with regulated behavior (Hardy et al., 2015; Malti and Dys, 2018; Crone and Fuligni, 2020). Moral identity may help students convert temporary compassionate states into more stable ethical self-guidance.

**FIGURE 1:**
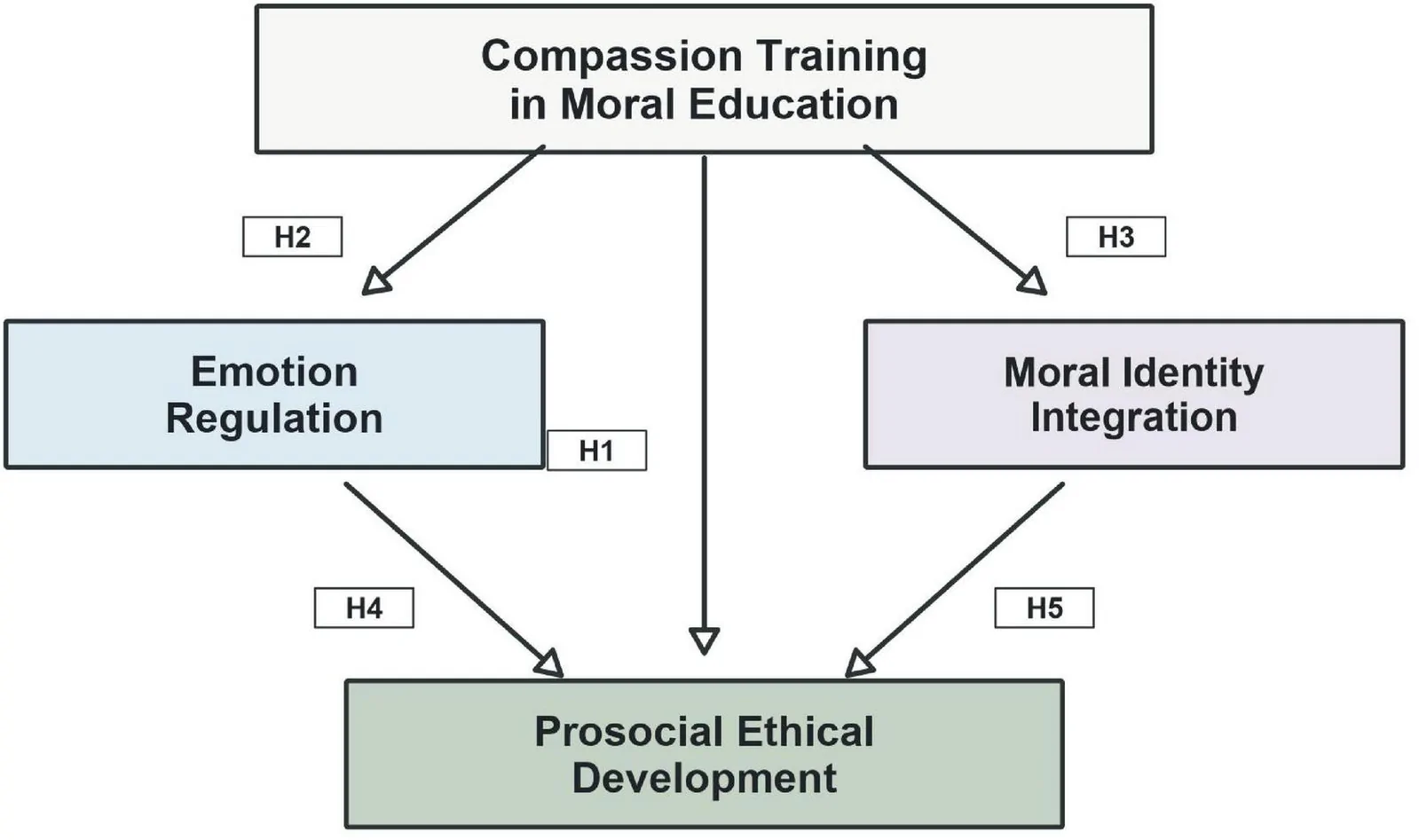
Conceptual framework linking compassion training, emotion regulation, moral identity integration, and prosocial ethical development.

Although compassion-based interventions have been studied in clinical, contemplative, and organizational settings, less is known about their longitudinal role in university moral education. Previous investigations have predominantly relied on cross-sectional designs or lacked rigorous baseline controls, severely limiting inferences regarding developmental trajectories. Furthermore, traditional moral education often approaches ethical learning as a purely cognitive load. By explicitly applying evolutionary psychology, the present study uniquely conceptualizes moral learning not merely as the rote memorization of rules, but as the activation of deeply conserved biological adaptations for social cooperation. This theoretical shift bridges the gap between biological predispositions and educational practice. Existing work also tends to examine compassion, emotion regulation, or prosocial behavior separately rather than modeling how these constructs operate together across time. The present study addresses this gap by testing a longitudinal mediation model in which compassion-training engagement predicts prosocial ethical development directly and indirectly through emotion regulation and moral identity integration. Figure 1 presents the conceptual framework.

## 2 Materials and methods

### 2.1 Study design and analytical framework

This study used a longitudinal quantitative design across one 16-week academic semester. This specific timeframe was purposefully selected to capture significant developmental and situational transitions, as students progress from initial academic orientation through mid-semester collaborative challenges and ultimately face end-of-term evaluative pressures. Such a trajectory provides a dynamic ecological setting for observing fluctuations in emotion regulation and the maturation of prosocial behaviors. The analytical framework was grounded in applied evolutionary psychology and moral education theory. Compassion training was conceptualized as an educational intervention that activates affiliative care, reduces threat-reactive responses, and supports cooperative ethical action. Emotion regulation and moral identity integration were modeled as parallel mediators, and prosocial ethical development was specified as the primary outcome.

The proposed model examined both direct and indirect pathways, incorporating an autoregressive cross-lagged panel approach to establish temporal precedence. Specifically, the model tested whether cumulative compassion-training engagement assessed at the mid-semester point (T1) predicted prosocial ethical development at the endline (T2), while rigorously controlling for pre-existing baseline levels (T0) of all mediators and the outcome variable. The indirect pathways tested whether this association was transmitted through improved emotion regulation and stronger moral identity integration. Structural equation modeling was selected because it allows simultaneous estimation of multiple latent constructs, direct effects, indirect effects, and total effects.

### 2.2 Research context and participant identification

Participants were undergraduates enrolled in moral education modules within Sino-foreign cooperative university contexts in China. This specific educational environment was purposively selected as the research setting because students in these joint programs often encounter multilingual instruction, intercultural value negotiation, and strong demands for self-management and social cooperation. By conducting the intervention within this naturally demanding ecology, the study could evaluate prosocial development under realistic conditions of high cognitive and emotional load. These features make it a suitable setting for examining how compassion-based moral education may support emotion regulation and prosocial ethical development.

**FIGURE 2:**
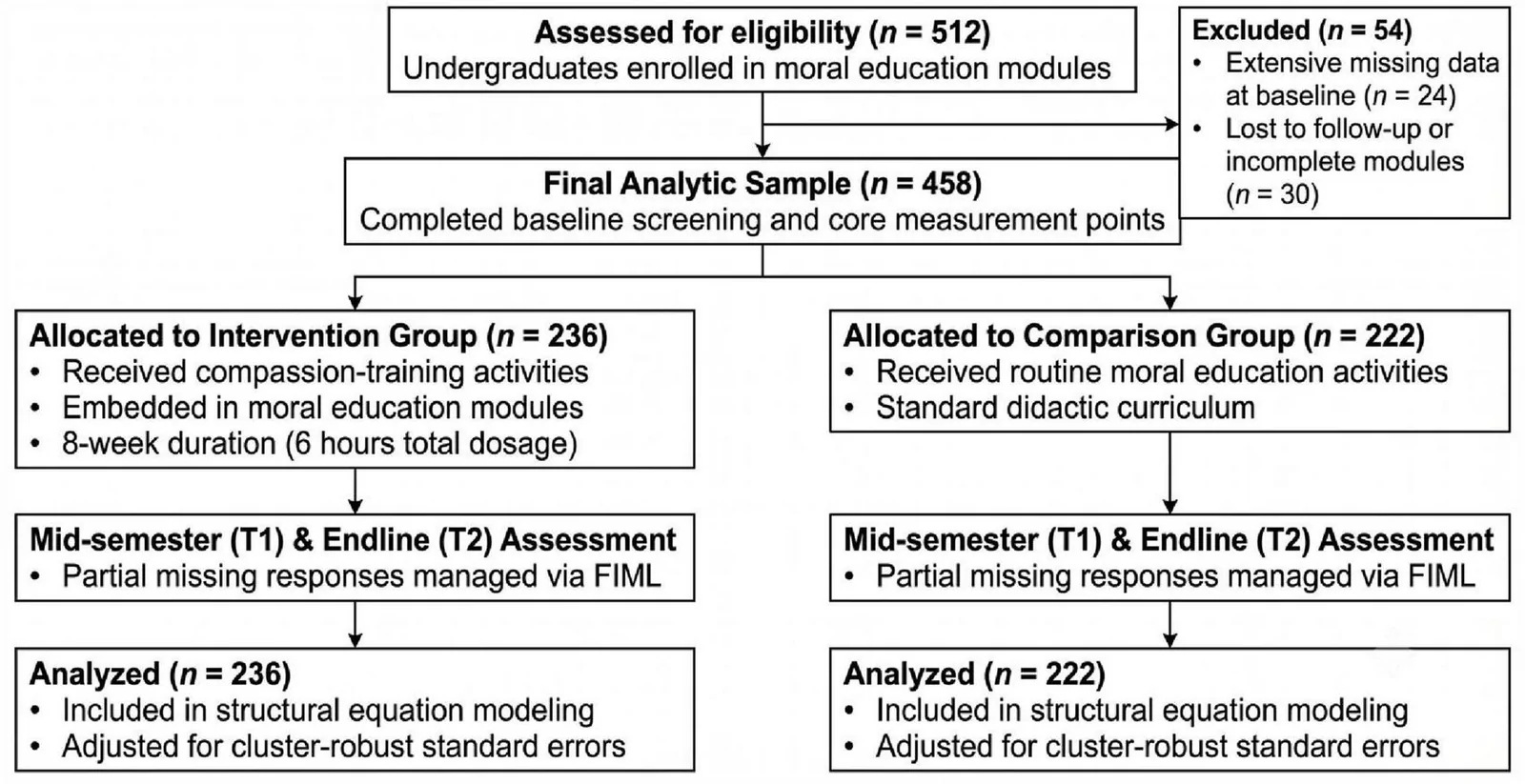
Participant flow diagram outlining the quasi-experimental study design. The diagram details the initial eligibility assessment, baseline exclusions, group allocation, and the final analytic sample subjected to structural equation modeling. Missing data across the longitudinal measurement points (T0, T1, T2) were handled using Full Information Maximum Likelihood (FIML).

Initially, 512 eligible students were assessed for inclusion (Figure 2). Following the baseline screening, 54 individuals were excluded, yielding a final analytic sample of 458 students. Students were screened through baseline moral education engagement, classroom participation, self-reported emotion-regulation difficulties, and indicators of low prosocial or ethical-action readiness. Inclusion criteria required formal undergraduate enrolment, completion of the course sequence during the semester, and valid responses at the core measurement points. Exclusions were strictly defined as either formal withdrawal from the moral education modules or failure to complete more than half of the assessment batteries across time points. Preliminary attrition analyses confirmed that dropout rates did not significantly differ between the intervention and comparison cohorts. To appropriately manage partial missing responses and minimize bias, the full information maximum likelihood procedure was applied during all structural estimations.

**FIGURE 3:**
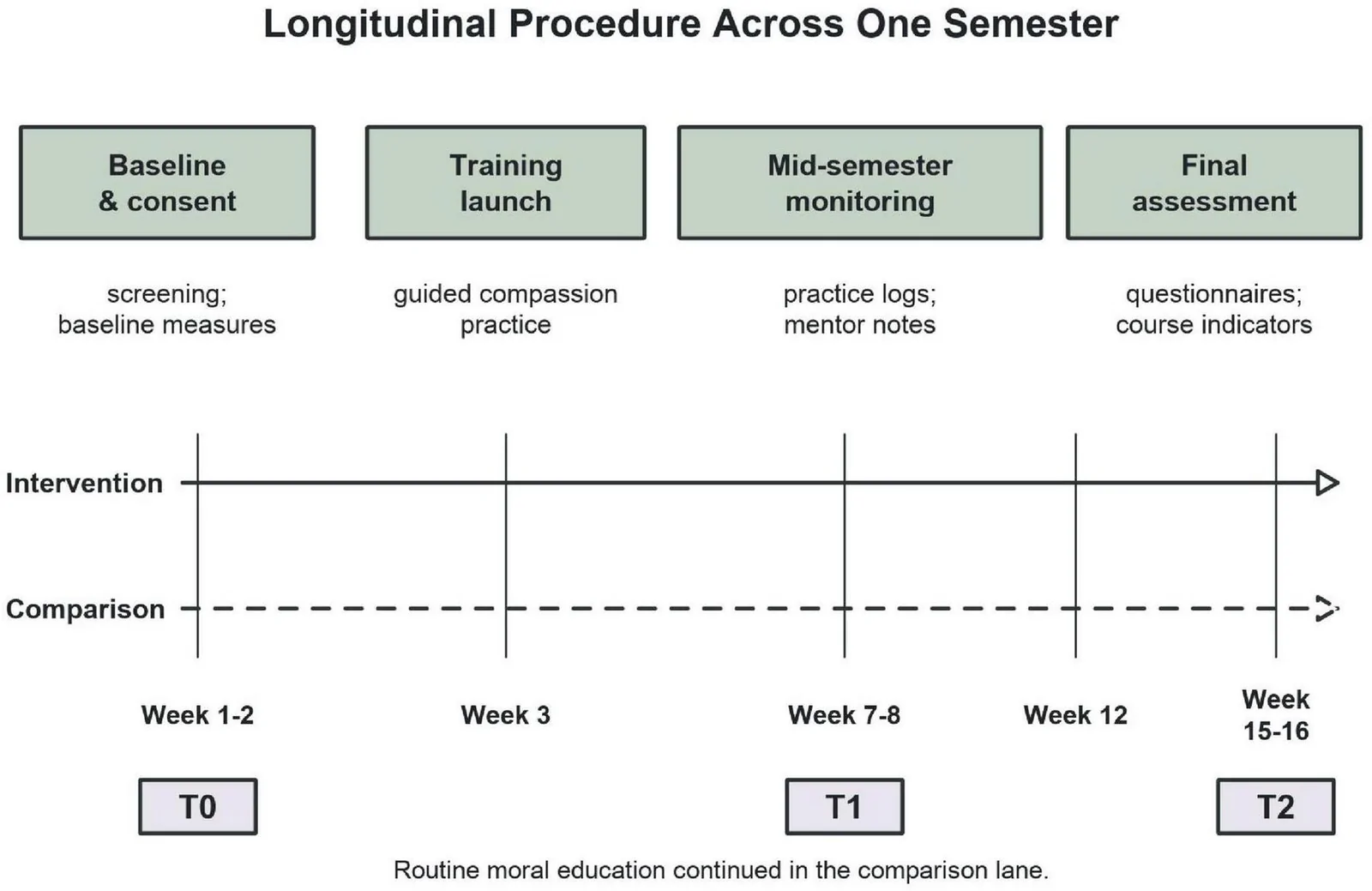
Timeline of participant identification, compassion-training implementation, comparison activities, and longitudinal data collection.

The longitudinal process followed five phases: baseline screening and consent, compassion-training launch, mid-semester monitoring, adaptive moral-education support, and final assessment. The intervention group received compassion-training activities integrated into the moral education course, whereas the comparison group received routine moral education activities and standard course resources. Groups were delineated based on pre-existing administrative classroom cohorts to preserve the naturalistic educational ecology and avoid cross-contamination of instructional methods. Although random assignment was not feasible, baseline equivalence was established; independent *t*-tests showed no significant group differences in baseline emotion regulation (*t* = 0.95, *p* = 0.34) or prosocial ethical development (*t* = 1.12, *p* = 0.26). Figure 3 summarizes the study timeline.

### 2.3 Compassion training intervention in moral education

The intervention was operationalized as a structured compassion-training component embedded in moral education. It drew on compassion cultivation and compassion-focused principles while adapting them to university classroom practice (Gilbert, 2014; Jazaieri et al., 2013; Neff and Germer, 2013). Because moral education is enacted through relationships as well as content, the design also drew on social-emotional learning research showing that teacher competence, classroom climate, and mindfulness-based school activities can support students’ social, emotional, and prosocial outcomes (Jennings and Greenberg, 2009; Schonert-Reichl et al., 2015). The course activities emphasized five dimensions: awareness of suffering, caring motivation, perspective-taking, emotion-regulation practice, and prosocial action planning.

Training activities included guided reflection, brief attention practices, discussion of moral dilemmas, perspective-taking exercises, peer dialogue, action planning, and reflective journaling. The intervention was delivered across eight consecutive weekly sessions, each lasting 45 min, culminating in a standardized total dosage of 6 h. These sessions followed a progressively scaffolded sequence designed to build psychological capacity incrementally. The initial 2 weeks established a foundational awareness of shared human vulnerability and suffering. The subsequent 3 weeks emphasized emotion regulation and active perspective-taking, utilizing concrete activities such as role-playing a marginalized peer during a simulated campus conflict to safely practice distress tolerance. The final 3 weeks concentrated on translating these internalized capacities into actionable prosocial planning. To minimize instructor-level variation and guarantee implementation fidelity, all participating teachers underwent a rigorous 2-day training seminar and adhered to a comprehensive pedagogical manual. Furthermore, independent fidelity checks were routinely executed via randomized classroom observations, confirming strict compliance with the instructional protocols across different teaching modules. Rather than treating compassion as an abstract virtue, the intervention framed it as a trainable social motivation that helps students regulate threat-based reactions and approach interpersonal problems with care. Teachers and mentors provided feedback on reflective tasks and supported transfer from classroom discussion to real-life ethical choices. In stark contrast to this experiential approach, the comparison group engaged exclusively with routine moral education materials. Their curriculum was strictly limited to traditional didactic lectures covering normative ethical theories and macro-level moral principles, entirely devoid of the targeted psychological cultivation and behavioral practice that characterized the intervention condition.

Implementation was monitored through attendance, practice logs, mentor notes, and classroom participation records. These implementation records were used to interpret the quantitative results but were not treated as a separate outcome model.

### 2.4 Measures and variable operationalization

The revised measurement model included four constructs. All questionnaire items used a 5-point Likert scale ranging from 1 (strongly disagree) to 5 (strongly agree), with higher scores indicating stronger endorsement of the construct. Prior to structural evaluation, a confirmatory factor analysis (CFA) validated the measurement model across all time points. The four-factor measurement model demonstrated excellent fit (e.g., SRMR = 0.045, CFI = 0.96, TLI = 0.95). Standardized factor loadings for all retained items ranged from 0.68 to 0.89, establishing strong indicator reliability. Internal consistency and convergent validity were confirmed as Cronbach alpha and composite reliability (CR) for all constructs consistently exceeded 0.82, with Average Variance Extracted (AVE) values surpassing the 0.50 threshold. Furthermore, discriminant validity was rigorously established; all Heterotrait-Monotrait (HTMT) ratios remained well below the conservative 0.85 limit. Additionally, longitudinal measurement invariance (including configural, metric, and scalar invariance) was established across the three time points, verifying that the latent constructs carried identical semantic meaning throughout the semester. Where possible, self-report indicators were supplemented with course participation records, practice logs, and mentor notes.

Compassion-training engagement was assessed using an *ad hoc* 6-item scale adapted from standard program evaluation frameworks to represent students’ perceived exposure to and engagement with the intervention. A sample item is I actively applied perspective-taking skills discussed in class during my daily interactions. Emotion regulation captured students’ ability to notice, interpret, and modulate emotional responses in moral or interpersonal situations. This construct was measured using a validated 15-item short form adapted directly from the Difficulties in Emotion Regulation Scale developed by Gratz and Roemer (2004), drawing conceptually on emotion-regulation theory and the multidimensional assessment of emotion-regulation difficulties (Gross, 1998; Gratz and Roemer, 2004), featuring items such as When faced with a moral conflict, I can effectively manage my initial anger.

Moral identity integration represented the degree to which caring, fairness, responsibility, and ethical consistency were incorporated into students’ self-concept. This construct was operationalized via a 10-item instrument originally conceptualized and validated by Aquino and Reed (2002) for assessing moral identity internalization, prompting students to rate their agreement with statements like Being a compassionate person is an important part of who I am. Prosocial ethical development was strictly operationalized as a self-reported behavioral intention construct rather than an objective behavioral metric. It was assessed via a 12-item composite scale derived fundamentally from the Prosocial Tendencies Measure created by Carlo and Randall (2002), referring to improvement in helping intention, fairness judgment, ethical action, and reduced harmful conduct. While these dimensions capture distinct facets of moral functioning, they were aggregated into a single outcome variable because theoretically they share a common prosocial-action orientation driven by affiliative care. Empirically, preliminary analyses revealed high intercorrelations among these subscales, and a second-order CFA confirmed that a single overarching latent factor adequately captured the shared variance, justifying their synthesis. It was informed by prosocial tendency measurement and moral identity research (Carlo and Randall, 2002; Hertz and Krettenauer, 2016).

### 2.5 Data collection procedures

Data were collected at three time points. At T0, during weeks 1–2, students completed baseline questionnaires measuring emotion regulation, moral identity integration, prosocial ethical development, and demographic information. Baseline screening also incorporated course participation and moral education engagement records. In week 3, the compassion-training component was launched in the intervention group.

At T1, during weeks 7–8, students completed mid-semester measures of compassion-training engagement, emotion regulation, and moral identity integration. This mid-semester assessment served to capture the intermediate mediating mechanisms following the initial training phase, thereby establishing a clear chronological sequence prior to the final outcome evaluation. Teachers and mentors also reviewed practice logs and reflective learning notes. At T2, during weeks 15–16, final questionnaires specifically targeting prosocial ethical development and course-based indicators were collected, completing the longitudinal mediation sequence (T0 baseline covariates predicting T1 engagement and mediators, which subsequently predict T2 outcomes). The final dataset combined questionnaire responses, practice records, participation indicators, and mentor notes.

### 2.6 Data analysis strategy

Descriptive statistics and bivariate correlations were first calculated for the four main variables. Because the primary constructs relied heavily on self-reported questionnaires, strict analytical objectivity was maintained through rigorous statistical cross-validation and bias-detection protocols. Specifically, diagnostic procedures were conducted to evaluate common method variance. Harman s single-factor test and an unmeasured latent method construct analysis indicated that method bias did not substantially distort the observed relationships, ensuring that our subsequent interpretations remained empirically grounded rather than artifacts of subjective reporting. Group differences were then examined by gender, year of study, language environment of instruction, and baseline prosocial-development risk level. The structural model was estimated to test the direct association between compassion-training engagement (measured at T1) and prosocial ethical development (measured at T2), as well as the indirect effects through emotion regulation and moral identity integration (measured at T1). Crucially, baseline (T0) levels of the mediators and outcome were included as covariates to control for pre-existing differences and model true longitudinal change. To address the nested nature of the data (students nested within classrooms), cluster-robust standard errors were employed. Furthermore, Harman’s single-factor test and an unmeasured latent method construct (ULMC) approach confirmed that common method bias did not significantly distort the structural relationships.

Bootstrapping was used to estimate confidence intervals for indirect effects. Model evaluation included explained variance (*R*^2^), predictive relevance (*Q*^2^), local effect size (*f*^2^), and global fit indicators including SRMR, NFI, and RMS_theta. Statistical significance was interpreted at conventional thresholds, with *p* < 0.05 as the minimum criterion and *p* < 0.001 indicating stronger evidence. Additional documentation on variable coding, the analytical model structure, and model evaluation is provided in the Supplementary material.

## 3 Results

### 3.1 Descriptive statistics and bivariate correlations

Prior to structural testing, the measurement model was evaluated for reliability and validity. The confirmatory factor analysis demonstrated a robust fit to the data, with all standardized factor loadings exceeding 0.68. Internal consistency was confirmed as composite reliability values for all constructs ranged from 0.82 to 0.89, alongside Average Variance Extracted scores well above the 0.50 threshold. Furthermore, discriminant validity was supported because all Heterotrait-Monotrait ratios remained below 0.85. Table 1 reports descriptive statistics and bivariate correlations. The mean scores of compassion-training engagement, emotion regulation, moral identity integration, and prosocial ethical development were 3.47, 3.38, 3.29, and 3.41, respectively. Standard deviations ranged from 0.64 to 0.71, suggesting adequate variability across constructs.

**TABLE 1:** Descriptive statistics and correlations among compassion-training engagement, emotion regulation, moral identity integration, and prosocial ethical development.

Variable	M	SD	1	2	3	4
1. Compassion-training engagement	3.47	0.69	1	1	1	1
2. Emotion regulation	3.38	0.64	0.49***			
3. Moral identity integration	3.29	0.71	0.46***	0.43***		
4. Prosocial ethical development	3.41	0.67	0.52***	0.58***	0.55***	

M, mean; SD, standard deviation;

***p < 0.001.

All four variables were positively correlated. Compassion-training engagement was associated with emotion regulation (*r* = 0.49, *p* < 0.001), moral identity integration (*r* = 0.46, *p* < 0.001), and prosocial ethical development (*r* = 0.52, *p* < 0.001). Emotion regulation and moral identity integration were also strongly associated with prosocial ethical development (*r* = 0.58 and *r* = 0.55, respectively, both *p* < 0.001).

**FIGURE 4:**
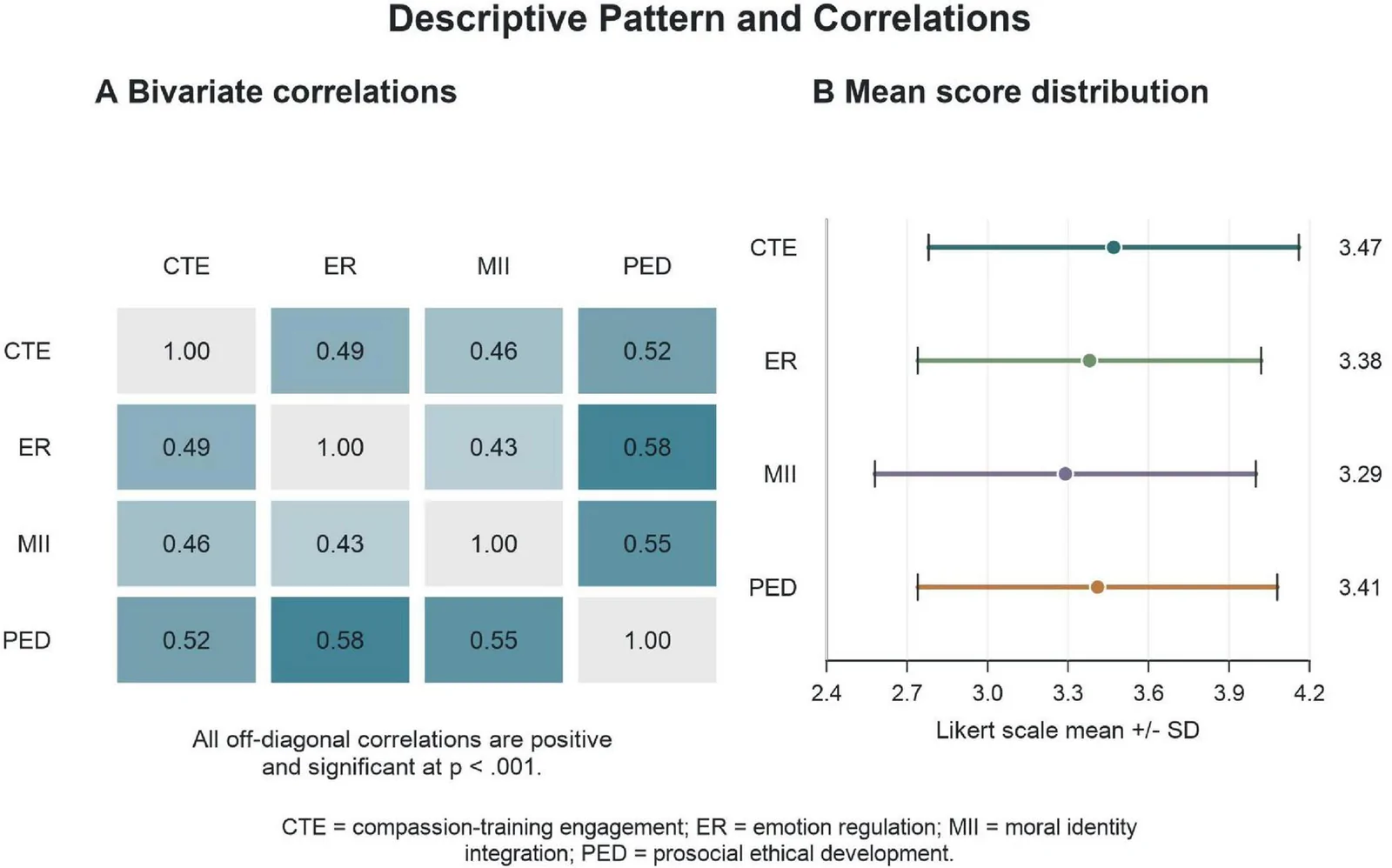
Descriptive mean scores, standard deviations, and bivariate correlations among the four primary study variables. **(A)** Bivariate correlation matrix; **(B)** means ± SD.

Figure 4 visualizes the same descriptive pattern by combining the correlation matrix with mean score distributions. The strongest bivariate association was observed between emotion regulation and prosocial ethical development, followed by moral identity integration and prosocial ethical development.

### 3.2 Group differences across student characteristics

Table 2 presents group differences across student characteristics. Female students reported higher scores than male students across the four constructs. Year of study showed a significant difference for compassion-training engagement, with lower-year students reporting somewhat stronger engagement. Language environment was most clearly associated with moral identity integration, suggesting that students in predominantly Chinese-medium settings may experience stronger alignment between classroom moral discourse and personal ethical identity. Baseline prosocial-development risk level showed the largest differences: moderate-risk students scored higher than high-risk students on all four constructs.

**TABLE 2:** Mean differences in the primary research variables across student subgroups.

Student characteristic	n	Training engagement, M ± SD	Emotion regulation, M ± SD	Moral identity, M ± SD	Prosocial development, M ± SD	Test statistic
Gender						
Male	214	3.39 ± 0.71	3.31 ± 0.66	3.22 ± 0.73	3.33 ± 0.69	
Female	244	3.54 ± 0.66	3.44 ± 0.61	3.35 ± 0.68	3.48 ± 0.64	
						*t* = 2.36*, 2.21*, 1.98*, 2.41*
Year of study						
Year 1	118	3.56 ± 0.65	3.42 ± 0.60	3.37 ± 0.67	3.49 ± 0.63	
Year 2	126	3.48 ± 0.68	3.39 ± 0.63	3.31 ± 0.70	3.43 ± 0.65	
Year 3	112	3.41 ± 0.71	3.35 ± 0.66	3.24 ± 0.72	3.38 ± 0.69	
Year 4	102	3.33 ± 0.72	3.33 ± 0.67	3.20 ± 0.75	3.31 ± 0.72	
						*F* = 2.84*, 0.96, 1.87, 2.31
Primary language of instruction						
Predominantly Chinese-medium	196	3.53 ± 0.67	3.42 ± 0.62	3.37 ± 0.69	3.46 ± 0.65	
Bilingual/English-intensive	262	3.42 ± 0.70	3.35 ± 0.65	3.23 ± 0.72	3.37 ± 0.68	
						*t* = 1.76, 1.21, 2.11*, 1.47
Baseline prosocial-development risk level						
Moderate risk	271	3.58 ± 0.63	3.46 ± 0.59	3.39 ± 0.66	3.52 ± 0.61	
High risk	187	3.31 ± 0.73	3.26 ± 0.69	3.15 ± 0.76	3.25 ± 0.73	
						*t* = 4.19***, 3.36**, 3.58***, 4.07***

M, mean; SD, standard deviation.

*p < 0.05;

**p < 0.01;

***p < 0.001.

### 3.3 Structural relationships among the main variables

Table 3 summarizes the structural path coefficients derived from the autoregressive cross-lagged panel model. After rigorously adjusting for baseline T0 covariate levels to isolate true longitudinal change over the semester, compassion-training engagement assessed at mid-semester T1 positively predicted emotion regulation at T1 (β = 0.41, *p* < 0.001), moral identity integration at T1 (β = 0.36, *p* < 0.001), and prosocial ethical development at the study endline T2 (β = 0.19, *p* < 0.001). Emotion regulation (β = 0.34, *p* < 0.001) and moral identity integration (β = 0.29, *p* < 0.001) both positively predicted prosocial ethical development.

**TABLE 3:** Structural paths linking compassion-training engagement, emotion regulation, moral identity integration, and prosocial ethical development.

Hypothesized path	Standardized β	SE	*T*-value	*P*-value	Result
Compassion-training engagement → emotion regulation	0.41	0.05	8.37	< 0.001	Supported
Compassion-training engagement → moral identity integration	0.36	0.05	7.24	< 0.001	Supported
Compassion-training engagement → prosocial ethical development	0.19	0.04	4.18	< 0.001	Supported
Emotion regulation → prosocial ethical development	0.34	0.05	6.62	< 0.001	Supported
Moral identity integration → prosocial ethical development	0.29	0.05	5.73	< 0.001	Supported

**FIGURE 5:**
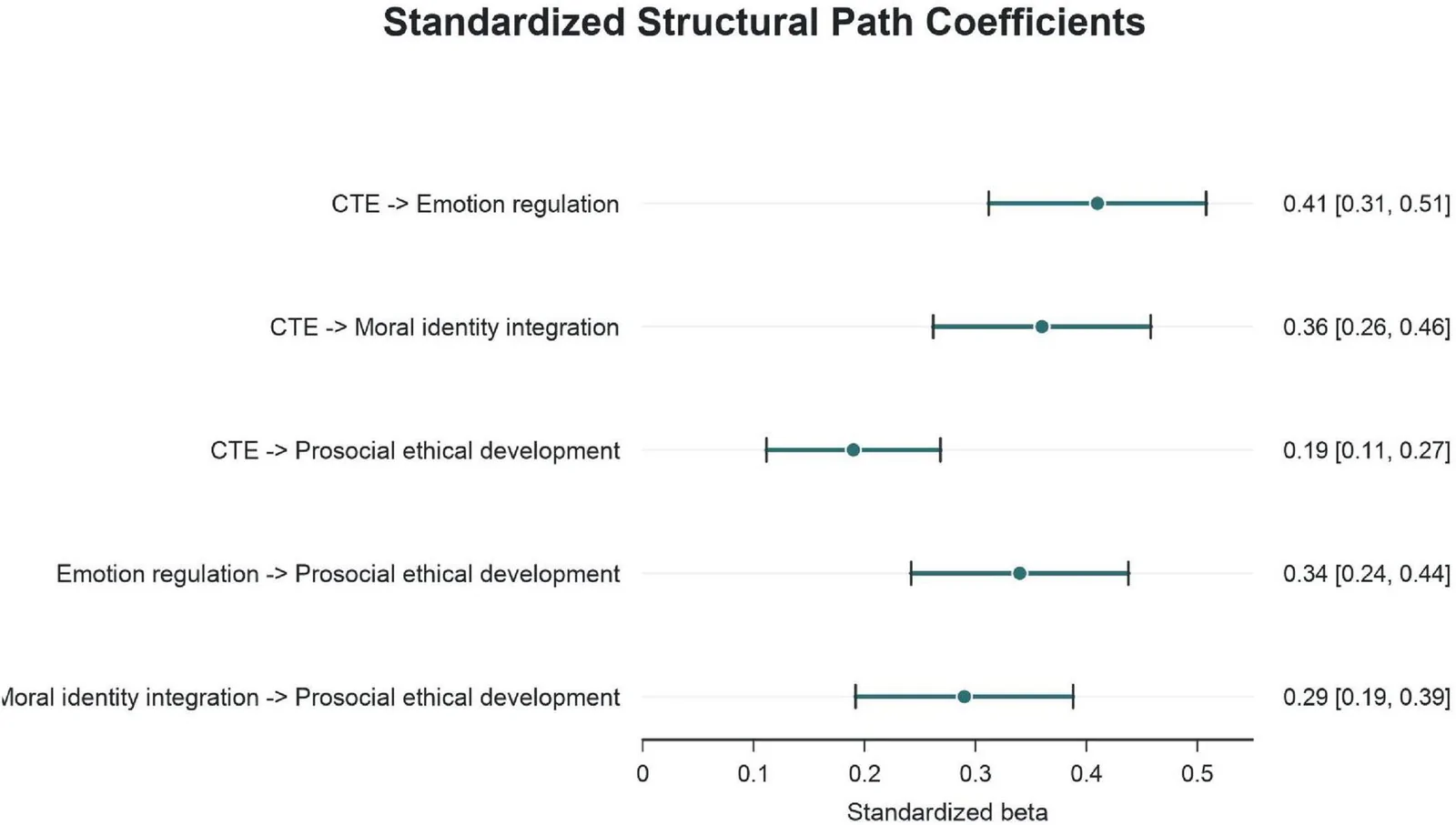
Forest plot of standardized structural path coefficients. Confidence intervals were approximated from the reported standard errors.

Figure 5 displays the standardized path coefficients as a forest plot. The strongest estimated path was from compassion-training engagement to emotion regulation, followed by compassion-training engagement to moral identity integration and emotion regulation to prosocial ethical development.

### 3.4 Mediation effects of emotion regulation and moral identity integration

Table 4 reports the mediation analysis testing the sequential pathways from baseline-adjusted T1 predictors to T2 outcomes. The indirect effect through T1 emotion regulation was 0.14 [95% CI (0.09, 0.20), *p* < 0.001]. The indirect effect through T1 moral identity integration was 0.10 [95% CI (0.06, 0.16], *p* < 0.001]. The total indirect effect was 0.24, while the direct effect remained significant (β = 0.19, *p* < 0.001), indicating partial mediation across the distinct temporal measurement points.

**TABLE 4:** Mediating roles of emotion regulation and moral identity integration in the association between compassion-training engagement and prosocial ethical development.

Path	Indirect effect (β)	SE	95% CI	*P*-value	Result
Training engagement → emotion regulation → prosocial ethical development	0.14	0.03	[0.09, 0.20]	< 0.001	Supported
Training engagement → moral identity integration → prosocial ethical development	0.10	0.03	[0.06, 0.16]	< 0.001	Supported
Total indirect effect	0.24	0.04	[0.17, 0.32]	< 0.001	Supported
Direct effect	0.19	0.04	[0.10, 0.28]	< 0.001	Supported
Total effect	0.43	0.05	[0.34, 0.53]	< 0.001	Supported

**FIGURE 6:**
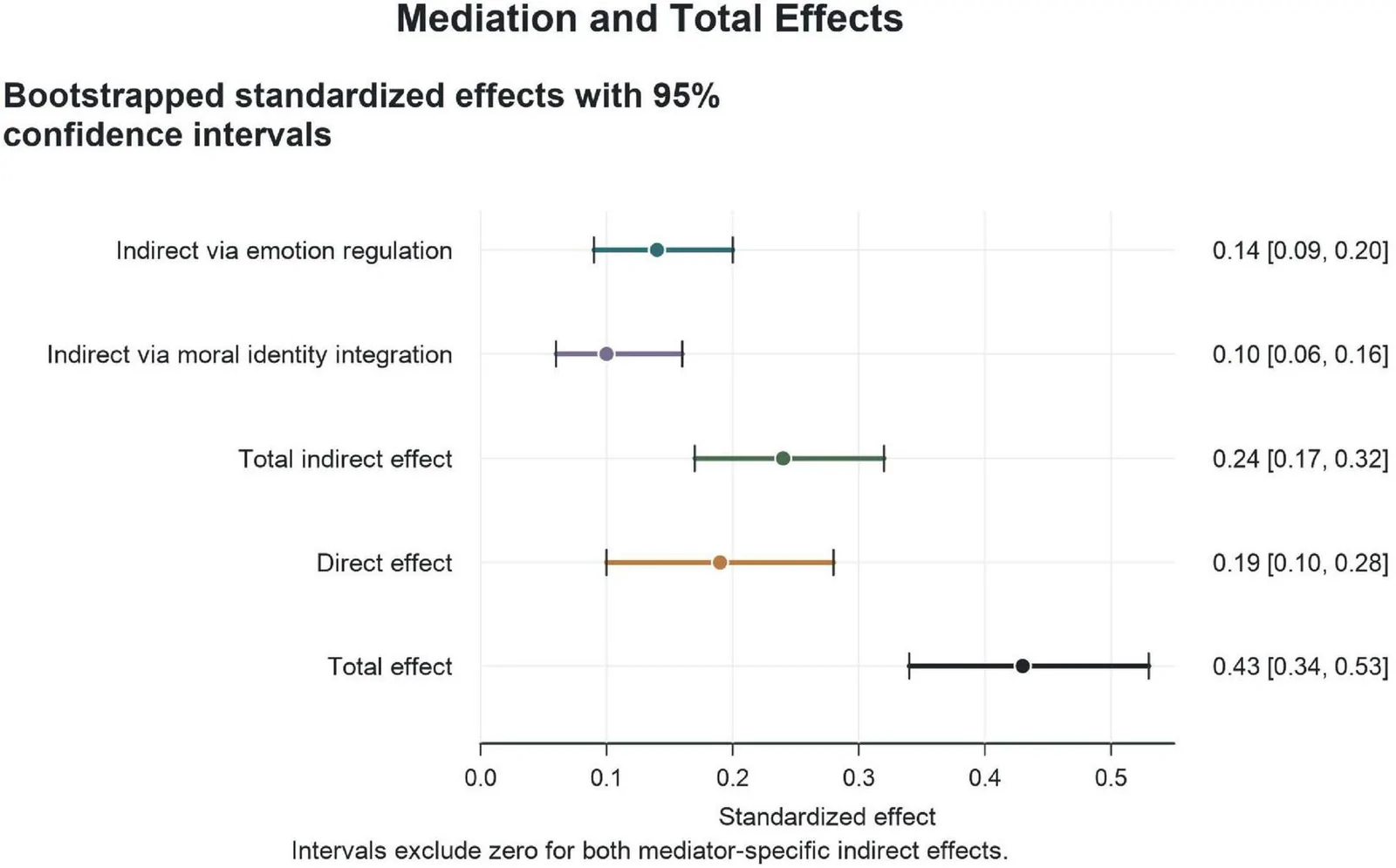
Bootstrap mediation, direct, indirect, and total-effect estimates with 95% confidence intervals.

Figure 6 presents the mediation estimates graphically. Both mediator-specific confidence intervals excluded zero, indicating that emotion regulation and moral identity integration each carried part of the association between compassion-training engagement and prosocial ethical development.

### 3.5 Model quality, predictive relevance, and integrated interpretation

The model showed adequate explanatory and predictive performance. The explained variance for prosocial ethical development was *R*^2^ = 0.46, indicating moderate-to-strong explanatory power. The *R*^2^ values for emotion regulation and moral identity integration were 0.17 and 0.13, respectively. All *Q*^2^ values were greater than zero, supporting predictive relevance.

Local effect sizes suggested that compassion-training engagement had medium effects on emotion regulation and moral identity integration, while its direct effect on prosocial ethical development was smaller. Emotion regulation had a somewhat stronger contribution to prosocial ethical development than moral identity integration. Overall fit was acceptable, with SRMR = 0.061, NFI = 0.91, and RMS_theta = 0.108.

**FIGURE 7:**
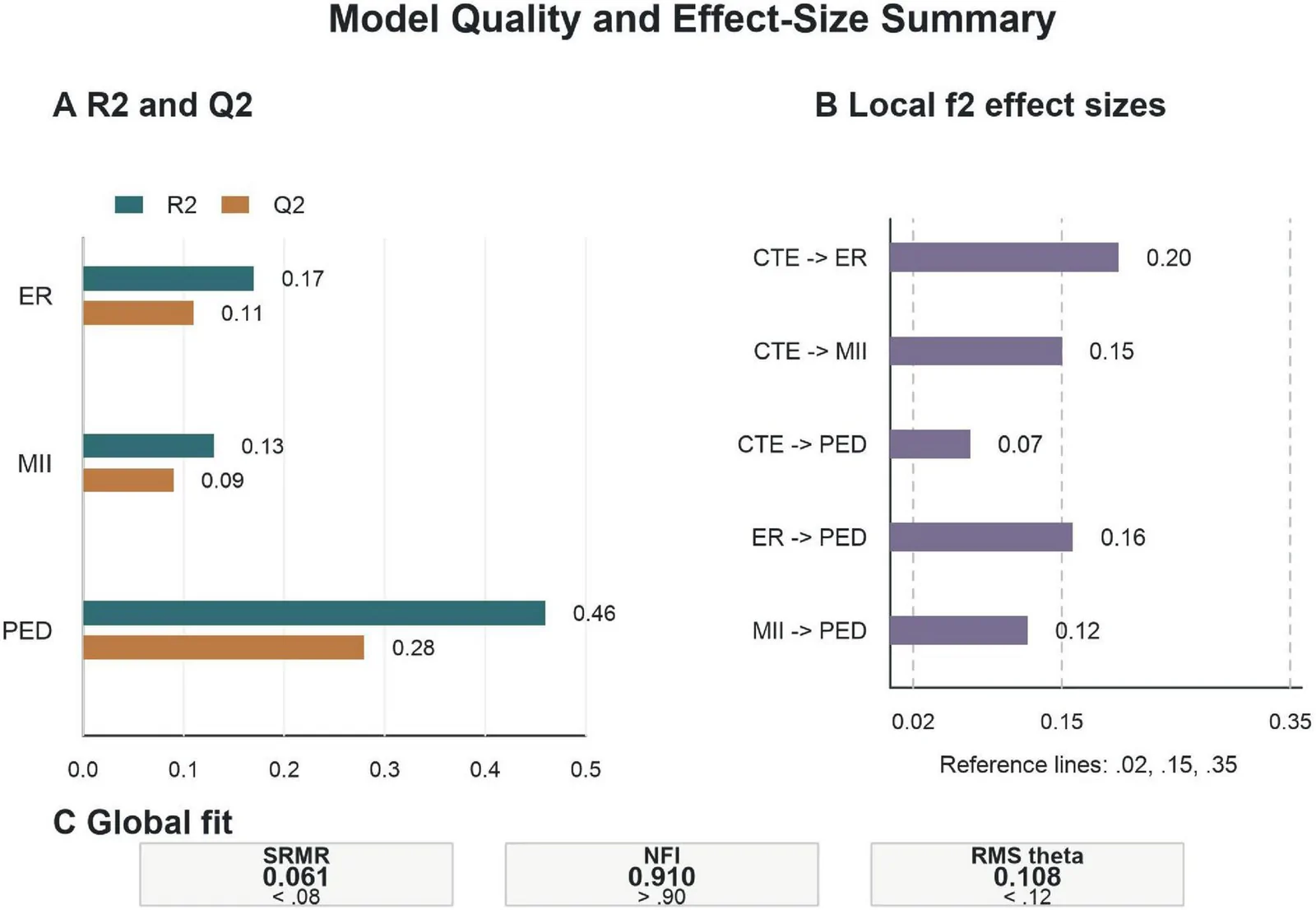
Model-quality summary showing **(A)** explained variance (*R*^2^) and predictive relevance (*Q*^2^), **(B)** local effect sizes (*f*^2^), and **(C)** global fit indices.

Figure 7 summarizes the model-quality evidence. Prosocial ethical development showed the strongest explained variance and predictive relevance, while the local effect sizes indicated that compassion-training engagement contributed most strongly to emotion regulation and moral identity integration.

## 4 Discussion

This study examined the longitudinal associations of compassion training in moral education, framing the analytical model primarily as an educational psychology investigation informed by theoretical accounts of affiliative care and compassion. The findings support the proposed model: compassion-training engagement was positively associated with prosocial ethical development, and this association was partially mediated by emotion regulation and moral identity integration. These results suggest that compassion training is longitudinally associated with strengthened moral education outcomes, potentially functioning by cultivating both affective regulation and a more stable ethical self-concept; nevertheless, the inherently correlational nature of the present research design inherently precludes the formulation of definitive causal claims. The model evaluation results, including explained variance (*R*^2^), predictive relevance (*Q*^2^), local effect sizes (*f*^2^), and model fit indices, are summarized in Table 5.

**TABLE 5:** Explained variance, predictive validity, effect sizes, and model fit indices.

Model evaluation index	Value	Recommended threshold	Interpretation
Explained variance (R2)			
Emotion regulation	0.17	> 0.10	Acceptable explanatory power
Moral identity integration	0.13	> 0.10	Acceptable explanatory power
Prosocial ethical development	0.46	> 0.25	Moderate-to-strong explanatory power
Predictive relevance (Q2)			
Emotion regulation	0.11	> 0	Predictive relevance established
Moral identity integration	0.09	> 0	Predictive relevance established
Prosocial ethical development	0.28	> 0	Good predictive relevance
Effect size (f2)			
Training engagement → emotion regulation	0.20	0.02/0.15/0.35	Medium effect
Training engagement → moral identity integration	0.15	0.02/0.15/0.35	Medium effect
Training engagement → prosocial ethical development	0.07	0.02/0.15/0.35	Small effect
Emotion regulation → prosocial ethical development	0.16	0.02/0.15/0.35	Medium effect
Moral identity integration → prosocial ethical development	0.12	0.02/0.15/0.35	Small-to-medium effect
Model fit indices			
SRMR	0.061	< 0.08	Good fit
NFI	0.91	> 0.90	Acceptable fit
RMS_theta	0.108	< 0.12	Acceptable fit

The first major finding is that compassion-training engagement directly predicted prosocial ethical development. This result is consistent with evolutionary accounts of compassion as an affiliative system oriented toward suffering, caregiving, and cooperative action (Goetz et al., 2010; Gilbert, 2014). While our empirical design relied on self-reports rather than directly measuring proximate evolutionary mechanisms (e.g., threat sensitivity or reciprocal altruism behavior), the findings align with the theoretical premise that educational practices can tap into these conserved social-motivational systems. In moral education, compassion training may help students move beyond abstract moral knowledge by practicing attention to suffering, perspective-taking, and concrete prosocial response planning. This is particularly important in university contexts where ethical choices often occur amid peer pressure, competition, and emotional reactivity.

The second major finding is the mediating role of emotion regulation. Compassion training was associated with stronger emotion regulation, and emotion regulation predicted prosocial ethical development. This pathway suggests that compassion does not simply increase emotional sensitivity; rather, it may help students manage distress, anger, shame, or avoidance in ways that make moral action more likely. This interpretation is consistent with emotion-regulation theory and prior compassion-training evidence showing reductions in worry and emotional suppression (Gross, 2015; Jazaieri et al., 2014).

The third major finding is the mediating role of moral identity integration. Students who reported stronger engagement with compassion training also reported a stronger integration of moral values into the self. Moral identity then predicted prosocial ethical development. This pathway is theoretically important because moral education aims not only to produce isolated prosocial acts but also to support durable ethical self-guidance. When compassion, fairness, and responsibility become part of students’ self-understanding, moral behavior may become more consistent across contexts.

The two mediating pathways were complementary. Emotion regulation explained how students manage affective responses in immediate moral situations; moral identity integration explained how moral commitments become more stable within the self. Together, these findings suggest that effective moral education should combine emotion-regulation practice with identity-level reflection. Compassion training is well suited to this task because it links awareness of suffering, caring motivation, reflective self-regulation, and action-oriented concern.

### 4.1 Implications for moral education

The results have several implications for university moral education. First, compassion training can be framed as a psychological skill rather than as a purely moral slogan. Activities such as guided reflection, perspective-taking, and emotion-labeling may help students regulate threat-based reactions before ethical decisions are made. Second, moral education should include opportunities for practice and transfer. Peer dialogue, mentor feedback, and ethical-action planning can help students connect classroom discussion to real behavior. This implication is consistent with evidence that classroom social-emotional conditions and mindfulness-based educational programs can influence prosocial and self-regulatory development (Jennings and Greenberg, 2009; Schonert-Reichl et al., 2015). Third, students with higher baseline prosocial-development risk may require more intensive support, because they showed lower scores across the core constructs.

### 4.2 Limitations and future research

Several limitations should be considered. First, the study relied predominantly on self-report measures, which may be affected by social desirability in moral education contexts. Although supplementary qualitative records such as practice logs and mentor notes informed the contextual interpretation of student engagement, they were not formally integrated into the quantitative structural models. Consequently, future research should include objective behavioral tasks, peer ratings, or teacher-blind evaluations of ethical action to systematically control for social desirability bias. Second, the intervention and comparison conditions were embedded in naturally occurring courses. Consequently, the capacity for robust causal inference remains strictly limited. The analytical framework, while longitudinally structured, essentially illustrates predictive associations rather than definitively establishing underlying psychological mechanisms. Without stringent experimental controls and true random assignment, the observed treatment outcomes cannot be entirely disentangled from pre-existing baseline engagement, inherent student motivation, or pervasive self-selection effects. Future investigations must deploy randomized controlled trials to systematically isolate the intervention impact. Third, the sample was drawn from Sino-foreign cooperative university contexts, and future studies should test whether the model generalizes to other institutions, age groups, and cultural settings. Finally, longer follow-up periods are needed to examine whether gains in emotion regulation and moral identity persist after the course ends.

## 5 Conclusion

This study provides a longitudinal model of compassion training in moral education from an applied evolutionary psychology perspective. Compassion-training engagement was associated with higher prosocial ethical development directly and indirectly through emotion regulation and moral identity integration. The findings suggest that moral education may be strengthened when it cultivates affiliative care, regulates threat-reactive emotions, and helps students integrate ethical commitments into the self. Compassion training therefore offers a promising associative link between moral knowledge, emotional capacity, and prosocial ethical action in higher education, warranting further rigorous experimental validation to confirm these dynamic pathways.

## Data Availability

The raw data supporting the conclusions of this article will be made available by the authors, without undue reservation.
